# The reliability and validity test of subjective cognitive decline questionnaire 21 with population in a Chinese community

**DOI:** 10.1002/brb3.2709

**Published:** 2022-07-21

**Authors:** Lixiao Hao, Jianguo Jia, Yue Xing, Ying Han

**Affiliations:** ^1^ Department of General Practice XuanWu Hospital of Capital Medical University Beijing China; ^2^ Department of General Surgery XuanWu Hospital of Capital Medical University Beijing China; ^3^ Department of Radiological Sciences, Division of Clinical Neuroscience, Queen's Medical Centre University of Nottingham Nottingham UK; ^4^ School of Biomedical Engineering Hainan University Haikou China; ^5^ Department of Neurology XuanWu Hospital of Capital Medical University Beijing China; ^6^ National Clinical Research Center for Geriatric Disorders XuanWu Hospital of Capital Medical University Beijing China; ^7^ Center of Alzheimer's Disease, Beijing Institute for Brain Disorders Beijing China

**Keywords:** Alzheimer's disease, discrimination, reliability, SCD‐Q21, validity

## Abstract

**Background:**

Subjective cognitive decline‐questionnaire 9 (SCD‐Q9) was developed to detect SCD complaints at risk of mild cognitive impairment (MCI). However, our previous findings indicated that its coverage might be insufficient. To test this hypothesis, we recently translated SCD‐Q21.

**Objective:**

To examine the reliability and validity of this translated SCD‐Q21 and to explore its effectiveness for discriminating MCI from controls.

**Methods:**

Item analysis was performed to understand its item discrimination and homogeneity. The Cronbach's α and Spearman‐Brown's split‐half coefficients were calculated to test its reliability. The Kaiser–Meyer–Olkin (KMO) value, Bartlett's sphericity test, and exploratory factor analysis (EFA) were used to examine its construct validity. The content validity was evaluated using five‐grade Likert scale. Finally, the SCD‐Q21 scores in MCI and controls were compared.

**Results:**

The difference of each item between the extreme groups was significant. The Cronbach's α coefficient was .913 and Spearman‐Brown's split‐half coefficient was .894. When performing holding one‐out approach, the Cronbach's α coefficient ranged from .906 to .914. The KMO value was .929 and the difference of Bartlett's Sphericity test was significant. All experts scored 5 points when assessing its content. Finally, a significant difference of score was found between MCI and NC groups.

**Conclusions:**

The reliability and validity of the SCD‐Q21 are good, which may pave a way for its application in a wider Chinese‐speaking population.

## Graphical abstract

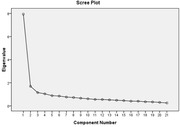

## 1 INTRODUCTION

World Alzheimer Reports showed that 47 million people lived with dementia worldwide in 2016 (Alzheimer's Disease International, 2016), and the number of patients with dementia in China accounts for approximately 25% of the entire population (GBD 2016 Dementia Collaborators, 2019), creating a huge challenge for policy makers, health‐care professionals, and family members (Jia et al., 2020). Till now, no drug trials have successfully demonstrated any effective treatment for Alzheimer's disease (AD) dementia (Doody et al., 2014). Mild cognitive impairment (MCI) may represent a transitional stage between healthy aging and dementia. Previous evidence based on a large population demonstrated that the percentage of progression from MCI to dementia ranged from 20% to 40%, and the annual conversion rates were 10%–15% (Roberts & Knopman, 2013). The failure of drug trials for AD treatment has shifted researchers' focus toward delaying progression from MCI to dementia (Anderson, 2019). In light of this purpose, it is important to detect early manifestation such as subjective cognitive decline (SCD) complaints in MCI for possible prevention or even modification of the progression of cognitive decline, especially in the communities where as many as 95% of elders report cognitive changes (Slavin et al., 2010). It is desirable to have a short screening and diagnostic tool that allows accurate measuring of SCD to alleviate the burden of patients and clinicians.

Different questions were used to investigate different aspects of the subjective cognitive changes in a few community‐based studies (Jessen et al., 2007; Lam et al., 2005). However, such variability in assessment methods might result in diverse conclusions (Abdulrab & Heun, 2008; Amariglio et al., 2011; Riedel‐Heller et al., 1999), and there was a need for a unified questionnaire that allows detection of MCI based on the SCD complaints. Gifford et al. (2015a) applied item response theory (IRT) and computerized adaptive test (CAT) methods to a questionnaire with initial 57 items related to MCI SCD complaints and condensed it into a potential tool for identifying MCI with only nine questions (SCD‐questionnaire 9, SCD‐Q9) (Cohen's *D* effect size = 0.49). Since its development, several studies (Alber et al., 2018; Bott et al., 2018; Kumar et al., 2018) have applied it to define SCD complaints or assess the changes of memory complaints. Our group translated it to Chinese and examined its validity and reliability. Our evidence demonstrated that the Chinese‐version of SCD‐Q9 has a good validity (see details in Supplementary Material S1). However, our results showed that SCD‐Q9 did not allow discrimination between MCI with SCD complaints and normal control (NC), and it was found that nearly 10% patients with MCI scored 0 (normal). One possible reason could be that the 9 items might not be sufficient to capture all the discriminable information of subjective complaints about cognitive decline in MCI. Additionally, Gifford et al. (2015a) reported that not all existing literature supports the final items included in SCD‐Q9 as some important aspects related to MCI might be neglected by using their selection approach (Gifford et al., 2015b). It is likely that the few more items included the SCD questionnaire 21 (SCD‐Q21) (Gifford et al., 2015a) may contain the information needed for detecting MCI patients.

With an increased prevalence or incidence of MCI in Chinese‐speaking populations around the world (approximately 14% prevalence of MCI in aged 60 years or older; Feng et al., 2019; Jia et al., 2020; Vanoh et al., 2017), it is of great importance to have a Chinese version of questionnaire inquiring SCD in MCI to study MCI in the Chinese communities. Based on such need, we translated the SCD‐Q21 (see details in Supplementary Material S2). To continue our previous work, our present study aimed to (1) test the reliability and validity of this translated SCD‐Q21 and (2) perform a preliminary analysis to investigate its discrimination power for MCI from NC in a Chinese community. Our ultimate aim is to widen the application of a reliable and short questionnaire for screening MCI with SCD complaints in the Chinese‐speaking population worldwide.

## 2 METHODS

### 2.1 Ethics statement

This study was approved by the Medical Ethics Committee of XuanWu Hospital of Capital Medical University, Beijing, China. Written informed consent was obtained from either participants or their legally agreed surrogates.

### 2.2 Participants

#### 2.2.1 Subject recruitment

This study obtained the support from the community of Fang Shan Guce District in Beijing, China. We recruited participants via advertising and broadcasting at large‐scale gatherings. Then, all of them were informed of the details of the study (e.g., its contact information, investigation purpose, and procedure) and asked to join the study voluntarily.

#### 2.2.2 Assessment procedure and study selection criteria

The recruitment was performed from July 1 to October 31, 2020. We used a convenience sampling to select the eligible individuals according to the inclusion and exclusion criteria shown below (see details in Supplementary Material Figure S1). All of subjects completed the questionnaires mainly based on self‐reporting, which includes sociodemographic information, medical history, and SCD‐Q21. And the entire procedure was performed by trained investigators with method of face‐to face interviews.

Inclusion criteria: long‐term residents (living in the target community for at least half a year), Han ethnicity, and ≥60 years old.

Exclusion criteria: (1) minority ethnics; (2) serious physical and mental illness(es); (3) had been diagnosed of dementia; (4) unable to participate in the study due to serious problems of hearing, speaking or vision.

Finally, all the eligible participants were asked to complete all the neuropsychological tests listed below.

#### 2.2.3 Assessment and diagnosis procedure

We collected information about sociodemography, medical history, and cognitive complaints and performed the general neurological examination for all the participants, which includes the sensory, motor responses, and reflexes. The Hamilton **Depression** (Hamilton, 1980) and **Anxiety** Scale (Tang & Zhang, 1984) were used to evaluate the up‐to‐date mood of participants. Subjects were also required to complete a battery of neuropsychological assessment tests containing four cognitive domains and global cognition function as follows: (1) Memory: Auditory Verbal Learning Test Hua Shan‐(AVLT‐H) (Guo et al., 2007); (2) Language: Animal Fluency Test (AFT) (Zhao et al., 2013a); (3) Executive: Trail Making Test B (STT‐B) (Zhao et al., 2013b); (4) Visual space: Clock Drawing Test (CDT‐30) (Guo et al., 2008); and (5) Global cognition function: Montreal Cognitive Assessment‐Basic (MoCA‐B) (Chen et al., 2016). In addition, we used the Clinical Dementia Rating Scale (CDR) (Morris, 1993) to assess the cognitive and functional performance related to the clinical stages of dementia. The social functioning was evaluated via Activity of Daily Living (ADL) (He et al., 1990). Finally, in order to differentiate degenerative and vascular etiologies, we performed the Hachinski Ischemic Index (HIS) (Hachinski et al., 1975) scale.

The MCI patients had to meet the following criteria (Petersen, 2004): (a) memory complaint, preferably confirmed by an informant (the answer needed to be “yes” to the question: “Do you have problem in memory?”); (b) objective memory impairment (scored at least 1.5 standard deviation [SD] below the norm in one or more cognitive fields, including global cognition, memory, executive function, language, or visuoconstructive skill); (c) near‐normal or normal general cognitive function performance with minimum or no impairment of daily life activities; (d) CDR score was 0.5; (e) HIS score less than or equal to 4; and (f) failure to meet the diagnostic criteria of dementia according to DSM‐IV (APA, 1994).

Criteria of NC was defined as follows: (a) having no memory complaints (the answer needed to be “no” to the question “Do you have problem in memory?”); (b) normal performance on global cognition function, memory, executive, visual space, and language with age‐ and education‐adjusted; (c) CDR scored 0; and (d) daily life activities were no impairment.

### 2.3 Statistical analysis

We used the Statistical Package for the Social Sciences version 17.0 (SPSS Inc., Chicago, IL) to conducted all analyses. Descriptive statistics for sociodemographic characteristics, and scores of SCD‐Q21 were presented as mean ± standard deviation (^−^ **x** ± *S*), percentages and median values. The Mann‐Whitney test or *t*‐test or χ^2^ was used to assess group differences, and *p *< .05 was considered to be statistically significant. Item analysis was applied to test the items discrimination and homogeneity. The difference of the extreme groups: higher scores group (top 27% with the highest scores) and lower scores group (bottom 27% with the lowest scores) (Liu et al., 2021) for each item was compared to test the discrimination. Spearman's correlation was conducted to correlate between each item and total scores of SCD‐Q21 to test the homogeneity; Cronbach's α and split half Spearman‐Brown coefficients were used to examine the questionnaire's internal consistency reliability. For examining the two validities: first, the content validity was evaluated by five‐grade Likert scale (high agreement: scored 5 points, agreement: 4 points, neutrality: 3 points, disagreement: 2 points and high disagreement: 1 points). Then, Kaiser–Meyer–Olkin (KMO) value, Bartlett's sphere test, and exploratory factor analysis (EFA) were used to assess the construct validity, and the gravel map was also draw to extract common factors. Finally, to better understand the discrimination power of SCD‐21 in detecting MCI, we compared the scores of SCD‐Q21 in NC and MCI groups, and the distribution histogram was displayed to present the total scores of the two groups.

## 3 RESULTS

### 3.1 Demographic characteristics of the participants

Two hundred and forty‐eight individuals with no dementia agreed to participate in our study, in which 87 (35.1%) were males, 161 (64.9%) were females, and the average age was 67.0 years of old (SD = 4.48). The mean education level was 6.81 ± 3.18 years.

### 3.2 Item analysis

#### 3.2.1 Item discrimination test

First, we conducted a comparison of each item between the higher scores and lower scores groups, and found that all the differences were significant (*p* < .001) (see Table 1).

**TABLE 1:** Comparison between higher and low groups of Chinese version of SCD‐Q21 items

	Group			
Items	Lower (*N* = 67) Percentile 50 (Percentile 25, 75)	Higher (*N* = 67) Percentile 50 (Percentile 25, 75)	Z	*p*
Q1	2.0 (2.0, 2.0)	1.0 (1.0, 1.0)	–10.174	<.001
Q2	2.0 (2.0, 2.0)	1.0 (1.0, 1.0)	–6.601	<.001
Q3	2.0 (2.0, 2.0)	1.0 (1.0, 1.0)	–10.300	<.001
Q4	3.0 (3.0, 3.0)	3.0 (2.0, 3.0)	–4.654	<.001
Q5	3.0 (3.0, 3.0)	3.0 (2.0, 3.0)	–4.460	<.001
Q6	2.0 (2.0, 2.0)	1.0 (1.0, 1.0)	–9.666	<.001
Q7	3.0 (2.0, 3.0)	2.0 (1.0, 2.0)	–7.297	<.001
Q8	2.0 (2.0, 2.0)	1.0 (1.0, 1.0)	–8.831	<.001
Q9	2.0 (2.0, 2.0)	1.0 (1.0, 1.0)	–9.124	<.001
Q10	3.0 (3.0, 3.0)	2.0 (1.0, 2.0)	–8.456	<.001
Q11	2.0 (2.0, 2.0)	1.0 (1.0, 1.0)	–8.338	<.001
Q12	2.0 (2.0, 2.0)	1.0 (1.0, 1.0)	–10.174	<.001
Q13	2.0 (2.0, 2.0)	2.0 (1.0, 2.0)	–6.193	<.001
Q14	2.0 (2.0, 2.0)	1.0 (1.0, 1.0)	–8.831	<.001
Q15	2.0 (2.0, 2.0)	1.0 (1.0, 1.0)	–9.124	<.001
Q16	2.0 (2.0, 2.0)	1.0 (1.0, 2.0)	–6.738	<.001
Q17	2.0 (2.0, 2.0)	1.0 (1.0, 2.0)	–7.898	<.001
Q18	2.0 (2.0, 2.0)	2.0 (1.0, 2.0)	–6.460	<.001
Q19	2.0 (2.0, 2.0)	1.0 (1.0, 1.0)	–9.471	<.001
Q20	3.0 (3.0, 3.0)	2.0 (1.0, 2.0)	–9.472	<.001
Q21	3.0 (2.0, 3.0)	2.0 (1.0, 2.0)	–7.336	<.001

*Note*: The number following SCD represents the question number of SCD‐Q 1 to 21.

#### 3.2.2 Item homogeneity test

The results of item homogeneity showed that there were significant negative correlations between each item and total scores of SCD‐Q21, and all the absolute values of correlation coefficients were above 0.35 with the highest in Q12 (*r* = −0.731) and lowest in Q5 (*r* = −0.352) (see Table 2).

**TABLE 2:** Correlation analysis between items and total scores of Chinese version of SCD‐Q21

Items	*r_s_*	*p*
Q1	–0.707	<.001
Q2	–0.496	<.001
Q3	–0.730	<.001
Q4	–0.380	<.001
Q5	–0.352	<.001
Q6	–0.648	<.001
Q7	–0.549	<.001
Q8	–0.651	<.001
Q9	–0.631	<.001
Q10	–0.597	<.001
Q11	–0.601	<.001
Q12	–0.731	<.001
Q13	–0.470	<.001
Q14	–0.660	<.001
Q15	–0.632	<.001
Q16	–0.495	<.001
Q17	–0.567	<.001
Q18	–0.450	<.001
Q19	–0.682	<.001
Q20	–0.686	<.001
Q21	–0.561	<.001

*Note*: The number following SCD represents the question number of SCD‐Q 1 to 21.

### 3.3 Reliability and validity test

#### 3.3.1 Reliability test

The test for internal consistency reliability demonstrated that the calculated Cronbach's α coefficient was .913 and Spearman‐Brown coefficient was .894. When holding each item of SCD‐Q21 out, the recalculated Cronbach's α coefficients ranged from .906 to .914 (see Table 3).

**TABLE 3:** Changes in Cronbach's α coefficients after deletion of Chinese version of SCD‐Q21 items

Items	Scale mean if item deleted	Scale variance if item deleted	Corrected item‐total correlation	Cronbach's alpha if item deleted
Q1	36.94	38.410	.617	.908
Q2	36.52	39.724	.466	.911
Q3	36.89	38.138	.635	.907
Q4	35.54	39.084	.438	.912
Q5	35.58	39.240	.372	.914
Q6	36.77	38.289	.612	.908
Q7	36.08	37.330	.560	.910
Q8	37.03	39.048	.548	.909
Q9	36.96	38.808	.554	.909
Q10	35.84	37.399	.582	.909
Q11	36.99	39.089	.517	.910
Q12	36.70	37.854	.702	.906
Q13	36.49	39.830	.477	.911
Q14	36.61	38.400	.649	.907
Q15	36.67	38.449	.610	.908
Q16	36.51	39.660	.495	.911
Q17	36.58	38.973	.565	.909
Q18	36.52	40.008	.414	.912
Q19	36.82	38.236	.620	.908
Q20	35.94	36.296	.690	.906
Q21	36.13	37.460	.568	.909

*Note*: **SCD‐Q21**: subjective cognitive decline‐questionnaire 21. **For SCD‐Q21,** the number following SCD represents the question number of SCD‐Q 1 to 21.

#### 3.3.2 Validity test

##### Content validity

A stringent content validity evaluation was conducted by five neurologists (four chief physicians and one deputy chief physician) coming from four cities (Beijing, Shanghai, Nanjing and Chongqing) in China. All of them are proficient in both Chinese and English and have over 10‐year experience in the diagnosis and treatment of AD. They appraised the equivalence, fluency and relevance of the content in relevant to the English version of SCD‐Q21, and discussed its expression to ensure a clear and unambiguous meaning of each item in each dimension. Finally, based on 5‐grade Likert scoring system, all of the experts reached a consensus (scored 5 points with high agreement) on the content of the Chinese version of SCD‐Q21.

##### Construct validity

**FIGURE 1:**
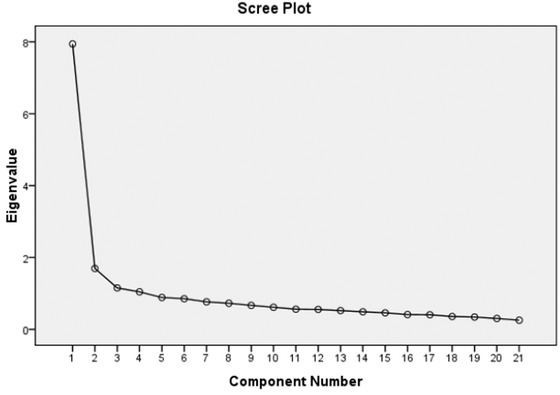
The gravel map of Chinese version of SCD‐Q21 by exploratory factor analysis. SCD‐Q21: subjective cognitive decline‐questionnaire 2

For construct validity, we found that KMO value was 0.929, the χ^2^ value of Bartlett's Sphericity test was 2092.686 and *p*<.001, As it is recommended that if the values of KMO were more than 0.600, further factor analysis is needed (Spicer, 2005). Thus we performed exploratory factor analysis. As a result, four common factors were extracted from the questionnaire, with eigenvalues of 7.943, 1.694, 1.152, and 1.043, respectively. The total cumulative contribution rate was 56.347% considering the four common factors (Figure 1). Detailed information for contribution rate and cumulative contribution rate of variance was presented in Table 4.

**TABLE 4:** Eigenvalue, variance contribution rate and cumulative variance contribution rate of common factors in Chinese version of SCD‐Q21

Component	Initial eigenvalues			Extraction sums of squared loadings			Rotation sums of squared loadings		
	Total	% of Variance	Cumulative %	Total	% of Variance	Cumulative %	Total	% of Variance	Cumulative %
1	7.943	37.824	37.824	7.943	37.824	37.824	3.864	18.400	18.400
2	1.694	8.067	45.891	1.694	8.067	45.891	3.168	15.087	33.488
3	1.152	5.487	51.379	1.152	5.487	51.379	3.156	15.031	48.519
4	1.043	4.969	56.347	1.043	4.969	56.347	1.644	7.829	56.347
5	0.886	4.218	60.566						
6	0.853	4.061	64.626						
7	0.765	3.641	68.267						
8	0.725	3.453	71.720						
9	0.667	3.176	74.896						
10	0.614	2.924	77.821						
11	0.559	2.664	80.485						
12	0.554	2.639	83.124						
13	0.522	2.484	85.608						
14	0.489	2.327	87.935						
15	0.460	2.188	90.124						
16	0.411	1.957	92.081						
17	0.407	1.938	94.019						
18	0.357	1.699	95.718						
19	0.343	1.631	97.349						
20	0.303	1.442	98.791						
21	0.254	1.209	100.000						

*Note*: **SCD‐Q21**: subjective cognitive decline‐questionnaire 21. For **SCD‐Q21,** the number following SCD represents the question number of SCD‐Q 1 to 21.

In addition, the orthogonal rotation method with maximum variance was conducted, and items with the largest absolute value of factor loadings were grouped into one category. As a result, items “Do you have complaints about your memory in the last 2 years?”(3), “Do you think that your memory is worse than 5 years ago?”(8), “Overall, do you feel you can remember things as well as you used to?” (11), “Do you think you have problems with your memory?” (1), “On a whole, do you think that your memory is good or poor?” (19), “Has your memory changed significantly?” (12) and “Do you feel you are forgetting where things were placed?” (9) were classified as common factor 1, items “Do you have more trouble remembering things that have happened recently?” (15), “Do you have difficulty remembering a conversation from a few days ago?” (2), “How often is the following a problem for you: Knowing whether you've already told someone something?” (10), “Do you feel you are unable to recall the names of good friends?” (18), “On a whole, do you think that you have problems remembering things that you want to do or say?” (6), and “How often is the following a problem for you: Going to the store and forgetting what you wanted to buy?” (7) were grouped into common factor 2, items “Do you notice yourself repeating the same question or story?” (16), “Do you feel that you have more memory problems than most?” (13), “Do memory problems make it harder to complete tasks that used to be easy?” (14), “Do you lose objects more often than you did previously?” (17), “How often is the following a problem for you: Words?” (21), and “How often is the following a problem for you: Things people tell you.” (20) belonged to common factor 3, and items “How often is the following a problem for you: Phone numbers you use frequently?” (5) and “How often is the following a problem for you: Personal dates (e.g., birthdays)?” (4) to common factor 4, with each common factor representing one dimension of SCD complaints (see details in Table 5, Supplementary Material S3, and Discussion).

**TABLE 5:** Rotated component matrix of SCD‐Q21

Items	Component			
	1	2	3	4
Q1	.745	.291	.155	–.006
Q2	.149	.608	.051	.245
Q3	.781	.321	.116	.000
Q4	.047	.260	.219	.753
Q5	.098	.148	.136	.842
Q6	.363	.523	.270	.047
Q7	.252	.455	.375	.040
Q8	.772	.041	.228	.054
Q9	.456	.383	.234	.007
Q10	.094	.603	.471	–.048
Q11	.758	.042	.146	.137
Q12	.491	.328	.427	.218
Q13	.219	.016	.646	.169
Q14	.325	.259	.577	.224
Q15	.268	.609	.198	.238
Q16	.109	.044	.758	.188
Q17	.201	.324	.575	.030
Q18	.116	.600	.025	.212
Q19	.676	.255	.220	.103
Q20	.251	.517	.525	.077
Q21	.176	.426	.533	–.019

*Note*: SCD‐Q21: subjective cognitive decline‐questionnaire 21. The number following SCD represents the question number of SCD‐Q 1 to 21.

### 3.4 The scores of SCD‐Q21 in NC and MCI groups

**FIGURE 2:**
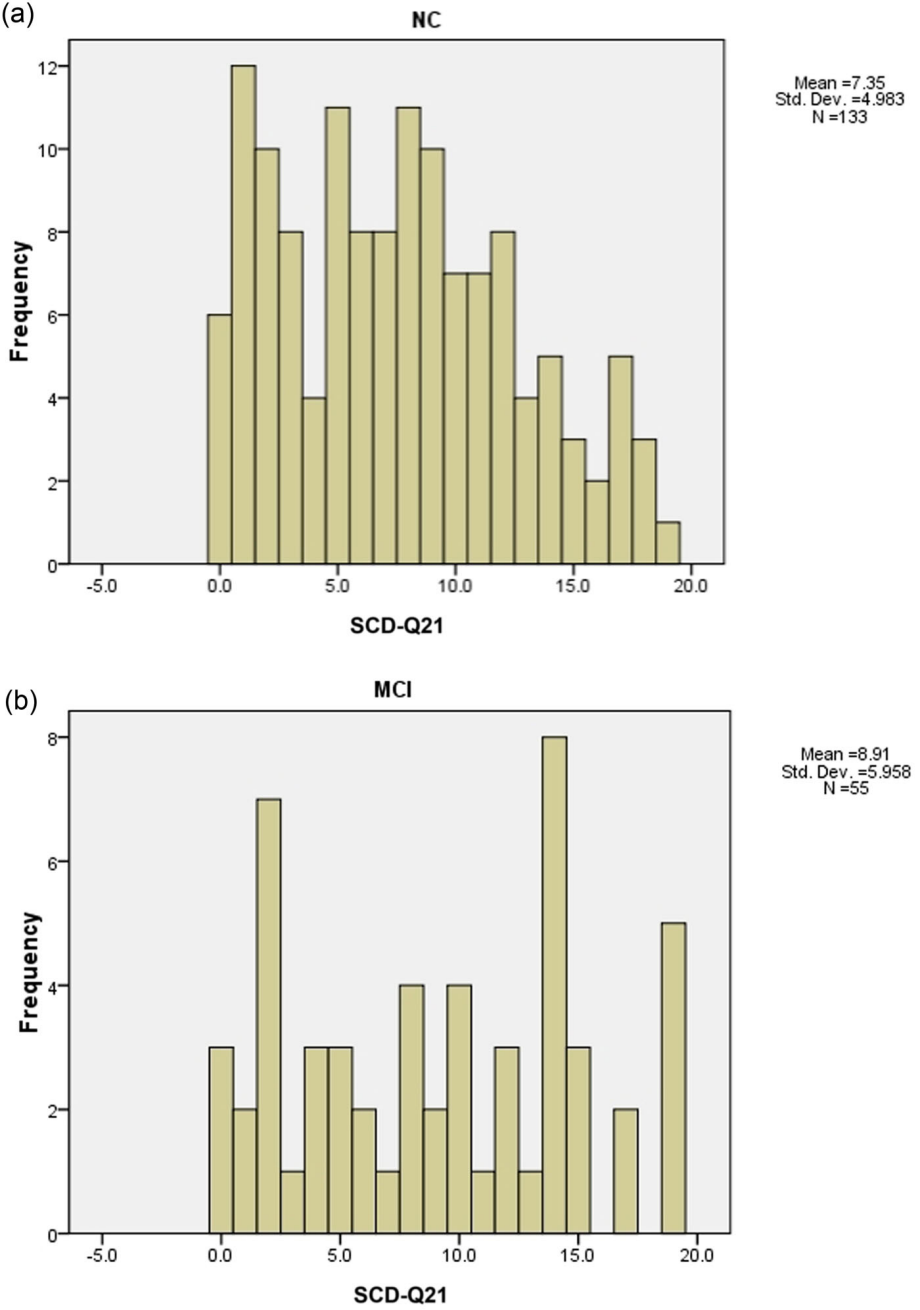
The frequency distribution histogram of SCD‐Q21 in NC and MCI groups. SCD‐Q21: subjective cognitive decline‐questionnaire **21**; NC: normal control, MCI: mild cognitive impairment

A total of 133 NC and 55 MCI were identified with completed the neuropsychological assessment tests in our cohort (clinical characteristics, including demography and scores of HAMA—an HAMD for NC and MCI groups; please see details in Supplementary Material Table S1 and Table 3). The distribution of SCD‐Q21 scores for NC and MCI groups was presented in Figure 2. By comparing the total scores SCD‐Q21 between NC (7.35 ± 4.98) and MCI (8.91 ± 5.96) groups, we found that MCI group scored significantly higher than the NC group (*p* < .05).

## 4 DISCUSSION

To our best knowledge, this is the first study attempting to test the reliability and validity of Chinese version of SCD‐Q21. Also, the present study performed a group comparison of its scores between MCI and NC groups with a Chinese‐speaking population.

The item analysis has been used to explore the differences between higher and lower score groups for each item or to test the homogeneity between items. It could also serve as a basis for item screening or modification (L M, 2010). In this study, the result of item analysis showed a good discrimination as well as a high homogeneity among all the items in SCD‐Q21, indicating a good score trend of this questionnaire and no items need to be removed.

The reliability test is mainly used to evaluate the accuracy, consistency, and stability of the questionnaire, which includes both the internal and external reliability. Cronbach's α and split half Spearman‐Brown coefficients are often used to examine the internal reliability. In our current study, the internal reliability of SCD‐Q21 was good, reflected by the high Cronbach's α and Spearman‐Brown coefficients according to Henson's recommendation (Henson, 2001). In addition, the Cronbach's α coefficient ranged from 0.906 to 0.914 with hold‐one‐out validation approach, suggesting each item of SCD‐Q21 contributed significantly to the reliability of the questionnaire. Smaller range fluctuations of the recalculated Cronbach's α coefficient also implies a good internal consistency of the questionnaire.

**The validity of the questionnaire means the authenticity and accuracy of its content**.

In our study, content validity was assessed by five experts to ensure that the translated version refers to the content of the original questionnaire appropriately while reflecting the cognitive characteristics (Shi et al., 2012). Construct validity is an important index to evaluate the quality of research tools. The sum of variance contribution rate of each factor is used as an index of evaluating the validity. Common factor 1 mainly queries about the global memory functioning and temporal comparisons. The second domain of SCD items queries about the individual's ability to complete daily or routine activities. Comparisons of memory with themselves and their peers are reflected by items in factor 3. The final domain of SCD items is related to the memory of important date and number (see details in Supplementary Material S3). The results of validity test showed SCD‐Q21 not only has good content validity, but also construct validity, although the total cumulative contribution rate was close to 60% (Spicer, 2005). The findings of construct validity in SCD‐Q21 were similar to SCD‐Q9 in common factor 1 and 2, and different from common factor 3 and 4. For instance, in the common factor 1, items “Do you think you have problems with your memory?” (1), “Do you have complaints about your memory in the last 2 years?” (3), “Do you think that your memory is worse than 5 years ago?” (8) and “Do you feel you are forgetting where things were placed?” (9) were in line with the English version of SCD‐Q9 (Gifford et al., 2015), but items “Overall, do you feel you can remember things as well as you used to?” (11), “Has your memory changed significantly?” (12) and “On a whole, do you think that your memory is good or poor?” (19) were newly added, which enquire about the global memory functioning. Similar to SCD‐Q9, items “Do you have difficulty remembering a conversation from a few days ago?” (2), “On a whole, do you think that you have problems remembering things that you want to do or say?” (6) and “How often is the following a problem for you: Going to the store and forgetting what you wanted to buy?” (7) were also identified, but several new items, such as items “How often is the following a problem for you: Knowing whether you've already told someone something?” (10), “Do you have more trouble remembering things that have happened recently?” (15), and “Do you feel you are unable to recall the names of good friends?” (18) of SCD‐Q21 were included in the common factor 2 in our study. Moreover, two new common factors were revealed in the current study. Common factor 3 reflected comparisons of memory with themselves and their peers (e.g., items “Do you feel that you have more memory problems than most?” (13), “Do memory problems make it harder to complete tasks that used to be easy?” (14), “Do you notice yourself repeating the same question or story?”(16), “Do you lose objects more often than you did previously?” (17), “How often is the following a problem for you: Things people tell you?” (20), and “How often is the following a problem for you: Words?” (21)), which has recommended as one important piece of diagnosis criteria of SCD (*plus*) by SCD‐Initiative (SCD‐I) (Jessen et al., 2014), yet there was no related information presented in SCD‐Q9. Finally, different from SCD‐Q9, items “How often is the following a problem for you: Personal dates (e.g., birthdays)?” (4) and “How often is the following a problem for you: Phone numbers you use frequently?” (5) in SCD‐Q21 reflect the ability to recall important things (date or number), which may imply a more advanced memory loss, involving pathological alterations (Amariglio et al., 2012).

Finally, we compared the scores of SCD‐Q21 in MCI and NC groups, and the result showed that MCI group had higher scores than that in the normal controls, indicating that SCD‐Q21 allowed distinguishing MCI from NC.

The limitations of this study include (1) the cumulative contribution rate in the current study was low, and less males participated our study compared with nonparticipants (*p* < .001) (please see details in the Supplementary Material Table S2), which may be due to our relatively small sample size. Further investigations with larger sample size are needed; (2) the test of reliability and validity was specific to our current cohort. This may vary for populations with different demographics. Moreover, the investigation site of our study is Fangshan District in Beijing, where most of individuals are Han ethnicity. To avoid the effect of the possible ethnicity on results (Burns et al., 2019), we included only individuals with Han ethnicity, which may limit the application in minorities. Therefore, future researches with a Chinese‐speaking population in different countries or cultural backgrounds (e.g., minorities, including Hui and Manchu, etc.) are needed to validate this finding; (3) an important next step would be investigating the link between SCD items identified in SCD‐Q21 to cognitive and neuroimaging evidence, and factors of unhealthy brain aging to further test the validity of this translated questionnaire; and (4) the nature of this questionnaire is subjective, and thus it may be subject to the individual perception and factors such as status mood and education level. Future studies are needed to investigate their effects. Finally, using CAT modeling to select the most commonly used items from SCD‐Q21 in the Chinese‐speaking population should be performed in our following research to directly compare SCD‐21 and SCD‐9.

In summary, we demonstrated a good reliability and validity of the Chinese version of SCD‐Q21. Also, the result of this preliminary analysis showed that SCD‐Q21 might allow screening MCI patients within the community population, which may pave a way for its application as a brief screening tool in a wider Chinese‐speaking population worldwide, but further investigation is needed for confirmation in the future.

## CONFLICT OF INTEREST

There is no conflict of interest.

### PEER REVIEW

The peer review history for this article is available at: https://publons.com/publon/10.1002/brb3.2709.

## Supporting information

Supplementary Information

Supplementary Information

Supplementary Information

Supplementary Information

Supplementary Information

Supplementary Information

Supplementary Information

## Data Availability

The data are confidential and not available.
