# Supplementary figures and images for: The reliability and validity test of subjective cognitive decline questionnaire 21 with population in a Chinese community
Source: Brain Behav. 2022 Jul 21;12(8):e2709. doi: 10.1002/brb3.2709 (PMC9392547; doi:10.1002/brb3.2709)

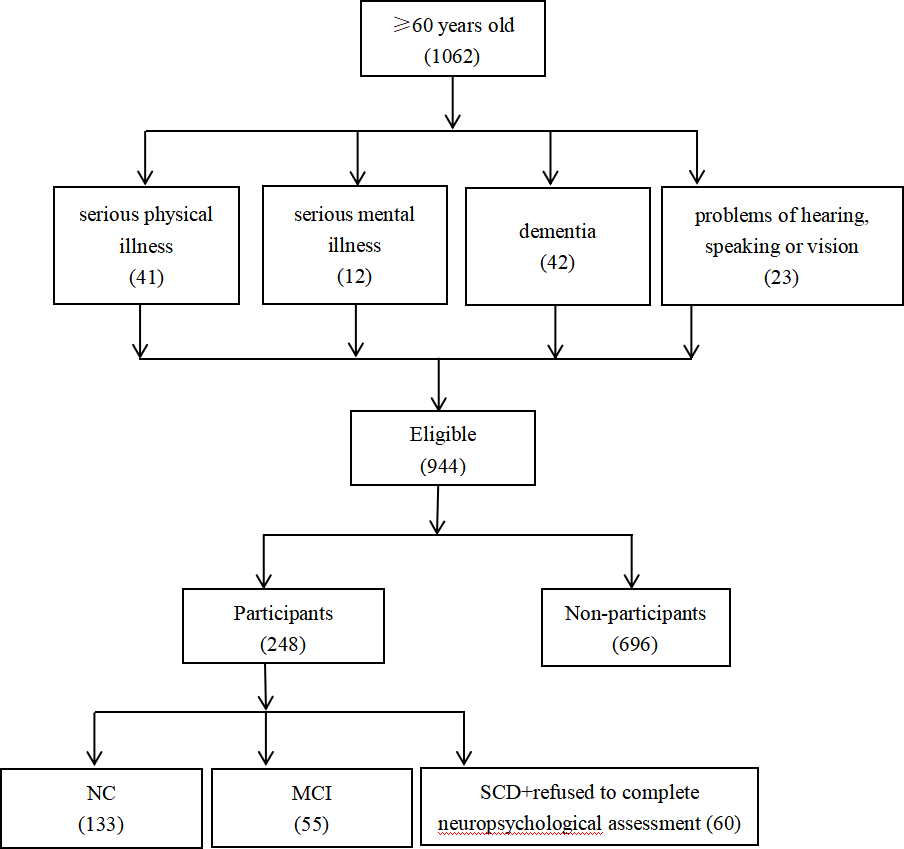

Supplement: Supplementary file 1 — Supplementary Information [file BRB3-12-e2709-s001.png]
