# Supplementary material for: The reliability and validity test of subjective cognitive decline questionnaire 21 with population in a Chinese community
Source: Brain Behav. 2022 Jul 21;12(8):e2709. doi: 10.1002/brb3.2709 (PMC9392547; doi:10.1002/brb3.2709)
Supplement: Supplementary file 2 — Supplementary Information [file BRB3-12-e2709-s002.docx]

Table S1. The scores of HAMA and HAMD in NC and MCI groups

|  | NC | MCI | *P* |
| --- | --- | --- | --- |
| HAMA | 2.0(0.5, 4.0) | 2.0(1.0, 6.0) | 0.068 |
| HAMD | 2.0(0.0, 4.0) | 1.0(0.0, 7.0) | 0.882 |

**NC:** Normal control; MCI: Mild cognitive impairment; **HAMA:** Hamilton anxiety scale; **HAMD:** Hamilton depression scale.

Table S2. The demographic characteristics of participants and non-participants

| Variables | Group | | *P* |
| --- | --- | --- | --- |
|  | Participants | Non-participants |  |
| Males, n (%) | 87(35.1) | 335(48.1) | ＜0.001 |
| Age, percentile50  (percentile 25, 75) | 67(63.25, 70) | 67(63, 72) | 0.103 |

Table S3. The demographic characteristics of NC and MCI groups

| Variables | Group | | *P* |
| --- | --- | --- | --- |
|  | NC | MCI |  |
| Males, n (%) | 51(38.3) | 17(30.9) | 0.405 |
| Age, ‾x±S | 66.23±4.34 | 67.16±4.50 | 0.821 |
| Education, ‾x±S | 7.38±3.06 | 6.62±3.03 | 0.912 |

**NC:** normal control; **MCI:** mild cognitive impairment.
