# Supplementary material for: The reliability and validity test of subjective cognitive decline questionnaire 21 with population in a Chinese community
Source: Brain Behav. 2022 Jul 21;12(8):e2709. doi: 10.1002/brb3.2709 (PMC9392547; doi:10.1002/brb3.2709)
Supplement: Supplementary file 3 — Supplementary Information [file BRB3-12-e2709-s003.docx]

Table 2. The demographic characteristics of participants and non-participants

| Variables | Group | | *P* |
| --- | --- | --- | --- |
|  | Participants | Non-participants |  |
| Males, n (%) | 87(35.1) | 335(48.1) | ＜0.001 |
| Age, percentile50  (percentile 25, 75) | 67(63.25, 70) | 67(63, 72) | 0.103 |
