# Supplementary material for: The reliability and validity test of subjective cognitive decline questionnaire 21 with population in a Chinese community
Source: Brain Behav. 2022 Jul 21;12(8):e2709. doi: 10.1002/brb3.2709 (PMC9392547; doi:10.1002/brb3.2709)
Supplement: Supplementary file 4 — Supplementary Information [file BRB3-12-e2709-s006.docx]

Table 3. The demographic characteristics of NC and MCI groups

| Variables | Group | | *P* |
| --- | --- | --- | --- |
|  | NC | MCI |  |
| Males, n (%) | 51(38.3) | 17(30.9) | 0.405 |
| Age, ‾x±S | 66.23±4.34 | 67.16±4.50 | 0.821 |
| Education, ‾x±S | 7.38±3.06 | 6.62±3.03 | 0.912 |

**NC:** normal control; **MCI:** mild cognitive impairment.
