# Supplementary material for: The reliability and validity test of subjective cognitive decline questionnaire 21 with population in a Chinese community
Source: Brain Behav. 2022 Jul 21;12(8):e2709. doi: 10.1002/brb3.2709 (PMC9392547; doi:10.1002/brb3.2709)
Supplement: Supplementary file 5 — Supplementary Information [file BRB3-12-e2709-s005.docx]

**Supplementary material 1**

**The reliability and validity test of subjective cognitive decline questionnaire9 (SCD-Q9)**

The Cronbach's α coefficient of SCD-Q9 was 0.886, after deleting any item of the questionnaire, the Cronbach's α coefficient ranged from 0.870 to 0.881. We also calculated the KMO value with 0.883. Combining the result of Bartlett's spherical test, our findings indicated that the questionnaire was suitable for further factor analysis. Finally, two common factors were extracted by exploratory factor analysis (EFA) with eigenvalue ≥ 1，whose cumulative variance contribution value was 70.519%(Hao et al., 2019).

**Reference**

Hao, L. X., Hu, X. C., Han, Y., & Jia, J. G. (2019). Localization of English Version of SCD-Q9 and

Reliability and validity Test, *Chinese General Practice*, 2019,22(26): 3238-3245.
