# Supplementary material for: The reliability and validity test of subjective cognitive decline questionnaire 21 with population in a Chinese community
Source: Brain Behav. 2022 Jul 21;12(8):e2709. doi: 10.1002/brb3.2709 (PMC9392547; doi:10.1002/brb3.2709)
Supplement: Supplementary file 6 — Supplementary Information [file BRB3-12-e2709-s007.docx]

**Translating English version of Subjective Cognitive Decline Questionnaire21 (SCD-Q21) to Chinese**

Our translated version of SCD-Q21 was under publication in the journal of Chinese General Practice(Hao et al., 2019)*.* We first obtained the permission from the authors of SCD-Q21(Gifford et al., 2015) and then followed the standardized process for translating foreign questionnaires into Chinese. The Brislin's model was used for translation and validation of instruments for cross-cultural research, including word-for-word translation, back- translation, and revision items for cultural adaptation, which were executed and agreed by a panel. This translated version was also tested with a small independent group of participants.

The English and Chinese version of SCD-Q21 see details as follows:

**Reference**

Hao, L. X., Xing, Y., Jia, J. G., & Han, Y., (2021). Localization of English Version of Subjective Cognitive Decline Questionnaire (SCD-Q21). *Chinese General Practice*, 2021,24(18): 2349-2354.

Gifford, K. A., Liu, D., Romano, R. R., Jones, R. N., & Jefferson, A. L. (2015). Development of a subjective cognitive decline questionnaire using item response theory: a pilot study. *Alzheimers Dement (Amst)*, 1(4), 429-439. http://doi.org/10.1016/j.dadm.2015.09.004

**English version of subjective cognitive decline**

**-questionnaire21 (SCD-Q21)**

1. Do you think you have problems with your memory?

1.Yes 2. No

1. Do you have difficulty remembering a conversation from a few days ago?

1.Yes 2.No

1. Do you have complaints about your memory in the last 2 years?

1.Yes 2.No

1. How often is the following a problem for you: Personal dates (e.g., birthdays)?

1.Always 2.Sometimes 3.Never

(5) How often is the following a problem for you: Phone numbers you use frequently?

1.Always 2.Sometimes 3.Never

(6) On a whole, do you think that you have problems remembering things that you want to do or say? 1.Yes 2.No

(7) How often is the following a problem for you: Going to the store and forgetting what you wanted to buy? 1.Always 2.Sometimes 3.Never

(8) Do you think that your memory is worse than 5 years ago?

1.Yes 2.No

(9) Do you feel you are forgetting where things were placed?

1.Yes 2.No

(10) How often is the following a problem for you: Knowing whether you’ve already told someone something 1.Always 2.Sometimes 3.Never

(11) Overall, do you feel you can remember things as well as you used to?

1.Yes 2. No

(12) Has your memory changed significantly?

1.Yes 2. No

(13) Do you feel that you have more memory problems than most?

1.Yes 2. No

(14) Do memory problems make it harder to complete tasks that used to be easy?

1.Yes 2. No

(15) Do you have more trouble remembering things that have happened recently?

1.Yes 2. No

(16) Do you notice yourself repeating the same question or story?

1.Yes 2. No

(17) Do you lose objects more often than you did previously?

1.Yes 2. No

(18) Do you feel you are unable to recall the names of good friends?

1.Yes 2. No

(19) On a whole, do you think that your memory is good or poor?

1.Good 2.Poor

(20) How often is the following a problem for you: Things people tell you

1.Always 2.Sometimes 3.Never

(21) How often is the following a problem for you: Words

1.Always 2.Sometimes 3.Never

**中文版主观认知下降问卷21（SCD-Q21）**

1. 你认为自己有记忆问题吗？

1.是，2.否

1. 你回忆3-5天前的对话有困难吗？

1.是，2.否

1. 你觉得自己近两年有记忆问题吗？

1.是，2.否

1. 下列问题经常发生吗：忘记对个人来说重要的日期（如生日）？

1.经常，2.偶尔，3.从未

1. 下列问题经常发生吗：忘记常用号码（如手机号、身份证号等）？

1.经常，2.偶尔，3.从未

1. 总的来说，你是否认为自己要做的事儿或要说的话容易忘记？

1.是，2.否

1. 下列问题经常发生吗：到了商店忘记要买什么？

1.经常，2.偶尔， 3.从未

1. 你认为自己的记忆力比5年前要差吗？

1.是，2.否

1. 你认为自己越来越记不住东西放哪儿了吗？

1.是，2.否

1. 以下问题经常发生吗：忘记你是否已经告诉某人某事？

1.经常，2.偶尔，3.从未

1. 总体而言，你是否觉得你的记忆力和以前一样好？

1.是，2.否

1. 你的记忆力有明显改变（下降）吗？

1.是，2.否

1. 你是否觉得你的记忆力比大多数人差？

1.是，2.否

1. 你的记忆力问题是否使你以前容易完成的任务现在变困难了？

1.是，2.否

1. 你是否更加记不住最近发生的事情？

1.是，2.否

1. 你是否注意到自己在重复讲述同样的问题或事情？

1.是，2.否

1. 你是否觉得比以前更常丢东西？

1.是，2.否

1. 你是否觉得你想不起好朋友的名字？

1.是，2.否

1. 总的来说，你认为你的记忆力是好还是差？

1.好，2.差

1. 以下问题经常发生吗：（忘记）别人告诉你的事情？

1.经常，2.偶尔，3.从未

1. 以下问题经常发生吗：找词困难？
2. 经常，2.偶尔，3.从未

**参考评分标准 二分类选项：是：1分；否：0分。**

**三分类选项：经常：1分；偶尔：0.5分；否：0分。**

**条目11和19为反向条目，其余为正向条目**

**总分为各条目相加得分。**
