# Supplementary material for: The reliability and validity test of subjective cognitive decline questionnaire 21 with population in a Chinese community
Source: Brain Behav. 2022 Jul 21;12(8):e2709. doi: 10.1002/brb3.2709 (PMC9392547; doi:10.1002/brb3.2709)
Supplement: Supplementary file 7 — Supplementary Information [file BRB3-12-e2709-s004.docx]

**Items sharing the same common factors**

**Common factor 1 (items 1, 3, 8, 9, 11, 12 and 19)**

Item **1** “ Do you think you have problems with your memory?” , item **3** “Do you have complaints about your memory in the last 2 years?”, item **8** “Do you think that your memory is worse than 5 years ago? ”, items **9** “Do you feel you are forgetting where things were placed?”, item **11** “Overall, do you feel you can remember things as well as you used to ?”, item **12** “Has your memory changed significantly?” and item **19** “On a whole, do you think that your memory is good or poor ?”.

**Common factor 2 (items 2, 6, 7, 10, 15 and 18)**

Item **2** “Do you have difficulty remembering a conversation from a few days ago? ”, item **6** “On a whole, do you think that you have problems remembering things that you want to do or say? ”, item **7** “How often is the following a problem for you: Going to the store and forgetting what you wanted to buy?”, item **10** “How often is the following a problem for you: Knowing whether you’ve already told someone something” , item **15** “Do you have more trouble remembering things that have happened recently? ” and item **18** “Do you feel you are unable to recall the names of good friends? ”.

**Common factor 3 (items 13, 14, 16, 17, 20 and 21)**

Item **13** “Do you feel that you have more memory problems than most? ”, item **14** “Do memory problems make it harder to complete tasks that used to be easy? ”, item **16** “Do you notice yourself repeating the same question or story? ”, item **17** “Do you lose objects more often than you did previously? ”, item **20** “How often is the following a problem for you: Things people tell you?” and item **21** “How often is the following a problem for you: Words”.

**Common factor 4 (items 4 and 5)**

Items **4** “How often is the following a problem for you: Personal dates (e.g., birthdays)?” and items **5** “How often is the following a problem for you: Phone numbers you use frequently? ”.
